# Stingless Bee Honey: Evaluating Its Antibacterial Activity and Bacterial Diversity

**DOI:** 10.3390/insects11080500

**Published:** 2020-08-04

**Authors:** Farah Nadiah Rosli, Mohd Hafiz Fikri Hazemi, Muhamad Afiq Akbar, Syazwani Basir, Hakimi Kassim, Hamidun Bunawan

**Affiliations:** 1 Centre for Biotechnology and Functional Food, Universiti Kebangsaan Malaysia, Bangi 43600, Selangor, Malaysia; farahnadiahrosli@gmail.com (F.N.R.); muhdafiq.akbar@gmail.com (M.A.A.); 2 Institute of Systems Biology, Universiti Kebangsaan Malaysia, Bangi 43600, Selangor, Malaysia; mohdhafizfikrihazemi@gmail.com (M.H.F.H.); syazwanibasir@gmail.com (S.B.); hakimikassim88@gmail.com (H.K.)

**Keywords:** stingless bee, honey, antimicrobial, bacterial diversity, amplicon sequencing

## Abstract

Stingless bee honey (SBH) is an astounding ‘miracle liquid’ with countless medicinal properties for various diseases such as gastroenteritis, cataracts, as well as for wound-healing. However, knowledge regarding it is still rather scarce. Henceforth, it is intriguing for us to contemplate on the less-studied stingless bee and its honey in particular. First and foremost, the antimicrobial ability of honey from eight different stingless bee species was tested to further proven its health benefit. *Homotrigona fimbriata* honey showed the highest antimicrobial activity with inhibition against five bacteria; *Serratia marcescens*, *Escherichia coli*, *Bacillus subtilis*, *Alcaligenes faecalis* and *Staphylococcus aureus*. The next aim of our study is to characterize their honey bacterial community via the use of 16S rRNA amplicon sequencing technology. A total of eight bacterial phyla, 71 families, 155 genera and 70 species were identified from our study and two of the stingless bee species honey were determined to have the highest bacterial diversity compared to other six stingless bee species, namely *Heterotrigona erythrogastra* and *Tetrigona melanoleuca*. Furthermost, *Lactobacillus malefermentans* was thought to be the native dominant bacteria of SBH due to its predominant presence throughout all studied species. The aforementioned SBH’s antimicrobial results and characterization study of its bacterial diversity are hoped to carve the pathway towards extending its probiotic ability into our everyday lives.

## 1. Introduction

Stingless bees—scientifically known as the tribe Meliponini—is the largest group of eusocial bees on Earth, with over 500 species—50 times more than its rival cousins, the honey bees (*Apis* sp.) [1,2]. They have inhabited earth 65 million years ago—longer than *Apis* sp.—and their honey has higher antimicrobial activity and less sugar content compared to most honey bees [3,4]. Yet far less research has been done on stingless bees than on honey bees [4]. Despite having countless medicinal properties, stingless bee honey has long been foreshadowed by the highly prevalent honey of *Apis* sp. which scientific studies have widely branched into diverse aspects such as behavioral science, microbiology, ecology, etc. In fact, most of our general knowledge on bees branched from an unrepresentative species, the western honey bee *Apis mellifera* [5]. The causal factor for this is partly due to the biologic manner of the stingless bee. Stingless bees are thought to produce less honey compared to honey bees, thus exhibiting less economic appeal to humans. This may have resulted from its lesser-known biologic manner and farming method [2].

SBH has long been consumed traditionally by humans for its nutritional and medicinal values [6]. It has been proven effective against diseases such as chemically induced cataracts, gastroenteritis and for wound-healing [4]. However, due to the paucity of knowledge regarding the product, SBH is not controlled by the food control authorities in some tropical countries such as Brazil [6]. Hence, the safety of the consumers are not guaranteed. Moreover, infants younger than twelve months are prohibited from being fed honey due to the possible present of botulism spores which can release dangerous toxins in a weak infant’s body, thus affecting their nervous system [7]. This limits the range of consumers for the nutritious honey. Moreover, stingless bee populations are in decline due to the increasing environmental deterioration and a large number of plants are greatly affected by the subsequent lack of pollination activity [8]. Therefore, an alternate way to gain health benefits from stingless bee honey without harming both the human and bees, and has high economical value is deprived to be contemplated.

One of the early steps toward finding the alternate way is via studying the antibacterial ability of stingless bee honey and characterizing its bacterial community. Bacteria have been known as the original source for important nutritional values such as probiotic in various food samples [9]. The probiotic ability of a substance is largely determined by its antimicrobial activity [10]. This activity could be partially due to the production of antimicrobials by bacteria present in probiotic foods. This would be the case for honey, as a past study has shown that 92.5% of total bacteria isolated from various floral honey manifest antimicrobial activity against at least one of their tested microbes [11]. Recently, most of the bacteria isolated from propolis of honey bees were demonstrated to exhibit a broad-spectrum antimicrobial activity against Gram-positive and Gram-negative bacteria and fungi [12]. The identification of stingless bee honey bacterial community and its antibacterial benefits could lead to its direct usage in probiotic production of food technology industry. Moreover, bacterial cultures provides a cheaper and higher production of probiotic compared to the stingless bees harvesting technique. However, it is important for researchers to differentiate between primarily acquired honey bacterial community such as bacteria from digestive tract of honeybees, nectar, pollen, propolis and flowers with secondarily acquired contamination from postharvest processing or appliances [13]. This is due to the pathogenicity potential of bacteria acquired from secondary sources unlike bacteria from primary sources which are more naturally obtained. The example of pathogenic bacteria acquired from secondary sources is *Clostridium botulinum* which is attained from contaminated water sources and ground. This pathogen is responsible for botulism disease among small children [13].

In this study, the antibacterial activity of stingless bee honey from eight different species was tested via agar well diffusion method against five different bacteria. Subsequently, 16S rRNA amplicon sequencing technology was used to characterize bacterial diversity of all eight stingless bee honey. This method could be regarded as an ideal bacterial screening method when compared to the conventional culturable method as it could identifies both culturable and unculturable bacteria [14]. A better characterization of honey bacterial community and its antibacterial potential will help to understand the possible role played by microbiota in the probiotic ability of stingless bee honey and many other important aspects of its biology. In addition, this investigation will expand the knowledge of how the honey bacterial community from stingless bee vary in different species aside from becoming a highlight due to the lack of research or report on the subject matter.

## 2. Materials and Methods

### 2.1. Sampling of Honey

Stingless bee honey samples were collected from TATI Agro Farm in Semenyih, Selangor approximately 25 kilometers from National University of Malaysia, Bangi. Honey samples were collected aseptically by using sterile sticks and sterile syringes before being transferred into sterile 15-mL Falcon tubes. Each sample contained 5 mL of honey and kept at 4 °C before further usage. Samples were chosen from eight different stingless bee species, namely A1, *Heterotrigona itama*; A2, *Heterotrigona erythrogastra*; B1, *Tetrigona apicalis*; B2, *Lepidotrigona terminata*; C1, *Tetrigona melanoleuca*; C2, *Tetrigona bingami*; D, *Geniotrigona thoracica*; E, *Homotrigona fimbriata* and labeled accordingly.

### 2.2. Antimicrobial Test

All eight different stingless bee honey species were diluted with sterile distilled water into three different concentrations which were 50%, 25% and 12.5%, each in triplicates. Next, antimicrobial activity of the diluted honey were tested against five different bacteria which are commonly used in evaluating honey antimicrobial activity comprising of two Gram-positive bacteria; Staphylococcus aureus (ATCC11632), Bacillus subtilis (ATCC11774) and three Gram-negative bacteria; Escherichia coli (ATCC10536), Serratia marcescens (ATCC13880) and Alcaligenes fecalis (ATCC15554) via agar well diffusion method using Mueller Hinton Agar (MHA) plates [15,16,17]. All bacteria were of standard strains (ATCC, US) and obtained from Microbiology Laboratory, Department of Bioscience and biotechnology, National University of Malaysia. A volume of 100 µl inoculum (10^7^–10^8^ CFU/mL) from each bacterium was spread on MHA plates and wells (7 mm diameter) were made in all inoculated plates before each being filled with 80 µl of the previously diluted honey samples. All prepared inoculated plates were incubated at 37 °C for 24 h. Sterile distilled water was used as negative control while ciprofloxacin antibiotic (30 µg/mL) was used as positive control. Finally, the diameter of inhibition zones were observed and measured. An inhibition zone with diameter ≥ 10 mm is considered as positive antagonism effect [18].

### 2.3. DNA Extraction

Each SBH sample consist of three biologic replicates acquired from different hives in the same farm. The pure honey samples obtained were diluted with sterile distilled water at ratio of 1:1, 15 mL of honey and 15 mL of sterile distilled water. Further filtration with 0.2-µM cellulose nitrate membrane was conducted on the diluted honey samples to filter the honey bacteria. Next, DNA extractions were carried out on each cellulose membrane with the acquired bacteria via CTAB extraction method by Doyle & Doyle and Cullings [19,20]. Three DNA samples of the same sample type were normalized to 50-ng DNA concentration and pooled together into one sample tube. The technique of pooling biologic replicates is commonly used in some Next Generation Sequencing studies [21].

### 2.4. PCR Amplification of 16S rRNA, Library Preparation and Sequencing

16S ribosomal RNA gene was amplified from 50 ng of genomic DNA using a bacteria-specific primer, 16S V3–V4 region with symmetric barcodes. The same barcode appears at both ends of an amplicon: 343F (5’-barcode-TACGGRAGGCAGCAG-3’) and 803R (5’-barcode- CTACCAGGGTATCTAATCC-3’). All PCR reactions were carried out with Phusion High-Fidelity PCR Master Mix (New England Biolabs). The conditions for PCR are as follows: initial denaturation at 94 °C (2 min), 30 cycles of denaturation at 94 °C (1 min), annealing at 60 °C (30 s), extension at 72 °C and final extension step of 10 min at 72 °C. Samples were run on 2% agarose gel for detection. Samples with bright main strip between 400–450 bp were chosen for further experiments. PCR products were purified with Qiagen gel extraction kit (Qiagen, Hilden, Germany). Sequencing libraries were generated using NEBNext Ultra DNA Library Pre®Kit for Illumina (New England Biolab, Ipswich, MS, USA), following manufacturer’s recommendations. The library quality was assessed on Agilent Bioanalyzer 2100 system. Lastly, the library was sequenced on an Illumina platform HiSeq 2500 and 250 bp paired-end reads were generated for each sample.

### 2.5. Sequence Data Analysis

Generated raw reads were processed via 4 stages to obtain clean effective tags. The first stage is paired-end reads assembly and quality control. In this stage, data split, sequence assembly, data filtration and chimera removal were executed using QIIME pipeline (version 1.70) and the FLASH tool (V1.2.7). Chimera removals were based on reference database (Gold database) by using the UCHIME algorithm. Clean effective tags generated from the first stage were then run on second stage which is OTU clustering and species annotation. Sequences with more than 97% similarity were assigned to the same OTUs via Uparse software (Uparsev7.0.1001) and representative sequences from each OTU were run on GreenGene database for annotation of species and its taxonomic information. In the third stage, statistical analysis of alpha diversity were carried out on the generated OTUs. Shannon and Simpson diversity indices were calculated using QIIME (version 1.70) and displayed with R software (version 2.15.3). In the fourth stage, statistical analysis of beta diversity were carried out on the generated OTUs which produced the principle coordination analysis, PCoA. This stage was also calculated using QIIME (version 1.70) and displayed with R software (version 2.15.3).

## 3. Results

### 3.1. Antimicrobial Activity

Antimicrobial results obtained from this study (Table 1) indicate that all honey samples with 50% concentration have positive antagonism effect against *S. aureus* except for *H. erythrogastra* honey sample which did not exhibit any inhibition zone. Meanwhile at 25% concentration, *T. apicalis*, *T. binghami*, *G. thoracica* and *H. erythrogastra* honey samples did not exhibit any inhibitory activity towards all five bacteria. At 12.5% concentration, only three honey samples showed inhibitory effect against one or more bacteria at the range of 10 to 16 mm in diameter of the inhibition zone. Collectively, *H. fimbriata* had the highest antimicrobial activity compared to other honey samples with inhibitory zone diameter up to 28 mm in size. On the contrary, *H. erythrogastra* had shown no inhibitory effect at all.

### 3.2. Sequencing Information and Bacterial Diversity of Stingless Bee Honey

A total of 703,348 reads were generated from eight honey samples of different stingless bee species, with an average of 12,839 sequences per sample for normalization. A total of 205 OTU bacteria was found in sample C1, *Tetrigona melanoleuca*, which is the highest among eight species. However, it falls behind at second to sample A2, *Heterotrigona erythrogastra* in terms of Shannon diversity index with a total of 158 OTU (Table 2). This indicates that honey produced from *Heterotrigona erythrogastra* has the highest bacterial species richness and evenness compared to other samples. Sample D, *Geniotrigona thoracica* has the lowest Shannon and Simpson diversity index with only 28 OTU bacteria. This indicates that honey produced from *Geniotrigona thoracica* has the lowest bacterial species richness and evenness compared to other samples. Meanwhile, sample B1, *Tetrigona apicalis* has the highest Simpson diversity index despite having the lowest number of OTU presents (Table 1). This however is not indicative towards high bacterial species richness and evenness and probably was influenced by the presence of low biomass level among samples. A significant difference could be seen among observed OTUs.

Based on rank abundance curve in Figure S1, sample C1 has the lowest level of steepness which directly indicates that it has the highest bacterial diversity. Meanwhile, sample B1 has the highest level of steepness which directly indicates that it has the lowest bacterial diversity. Although samples with the highest and lowest bacterial diversity varied between Shannon, Simpson diversity index (Table 1) and rank abundance curve (Figure S1), the variation is not far-off. Overall, samples A2, *Heterotrigona erythrogastra* and C1, *Tetrigona melanoleuca* are the top two for the highest bacterial diversity while samples D, *Geniotrigona thoracica* and B1, *Tetrigona apicalis* are the top two for the lowest bacterial diversity.

**Figure 1:**
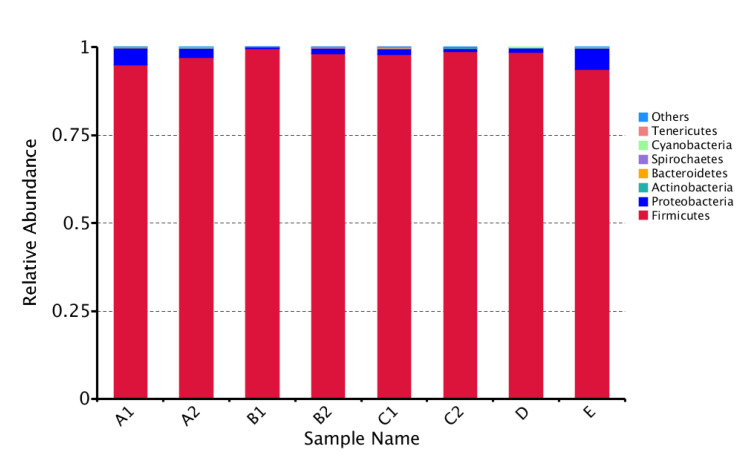
Bacterial phylum level composition in honey from eight different stingless bee species. Color-coded bar plot showing the average bacterial phylum distribution of the different groups sampled from honey of various stingless bee species (A1—*Heterotrigona itama*; A2—*Heterotrigona erythrogastra*; B1—*Tetrigona apicalis*; B2—*Lepidotrigona terminata*; C1—*Tetrigona melanoleuca*; C2—*Tetrigona bingami*; D—*Geniotrigona thoracica*; E—*Homotrigona fimbriata*).

In total, the OTUs were assigned to 8 bacterial phyla, 71 families, 155 genera and 70 species. At the phylum level, the single most abundant bacterial phyla is Firmicutes which are consistently predominant in all honey samples of eight stingless bee species with relative abundance close to 1.0 (Figure 1). There are no significant differences in the relative abundance of Firmicutes among different species which indicates the importance of bacteria from Firmicutes phylum in stingless bee honey.

Top seven OTU bacteria with high percentage abundance are shown in Table S1 and all are categorized under *Lactobacillus* genus. The most abundant OTU bacteria is OTU 1 which consists of *Lactobacillus malefermentans*. This bacterial species present abundantly in all samples and can be considered as the natively dominant bacteria in stingless bee honey. Other remaining top bacterial species are *Lactobacillus johnsonii*, *Lactobacillus wasatchensis*, *Lactobacillus amylovorus*, *Lactobacillus pentosiphilus* and *Lactobacillus salivarius*.

**Figure 2:**
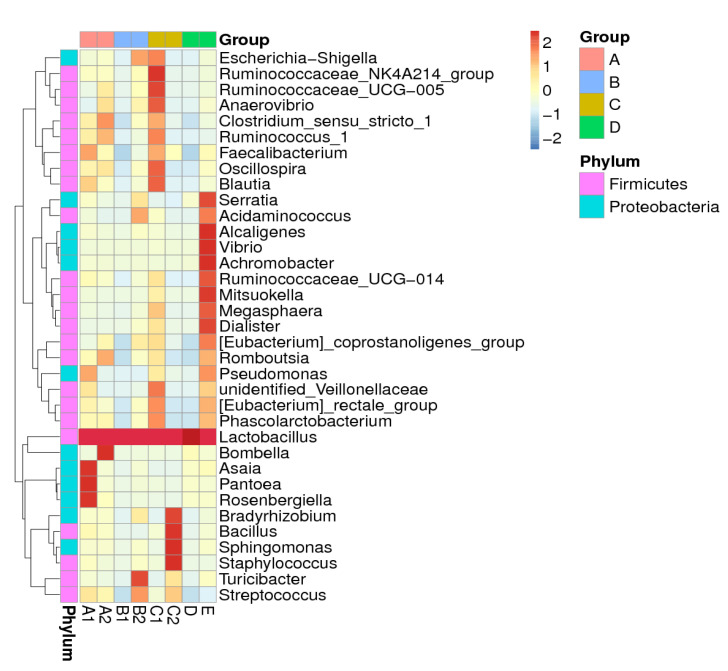
Heatmap showing 35 bacterial genus of stingless bee honey with significant differences in abundances between groups (Kruskal–Wallis test, *df* = 3, FDR-corrected *p* < 0.05).

At the genus level, 35 bacteria were found to have significant differences in abundance among all bacteria (Kruskal–Wallis test, *df* = 3, FDR-corrected, *p* < 0.05) as shown in Figure 2. Each sample species has different set of bacterial abundances (A1, 7 genus; A2, 5 genus; B1, 1 genus; B2, 6 genus; C1, 14 genus; C2, 5 genus; D, 1 genus; E, 16 genus). Sample E, *Homotrigona fimbriata* has the largest set of bacterial genus abundance which includes *Lactobacillus*, *Serratia*, *Alcaligenes*, *Vibrio*, *Achromobacter*, *Mitsuokella* and *Dialister*. Meanwhile, sample B1, *Tetrigona apicalis* and D, *Geniotrigona thoracica* has the smallest set of bacterial genus abundance which only includes *Lactobacillus*. There are no particular similar cluster of bacteria among samples and only *Lactobacillus* present abundantly in all samples.

**Figure 3:**
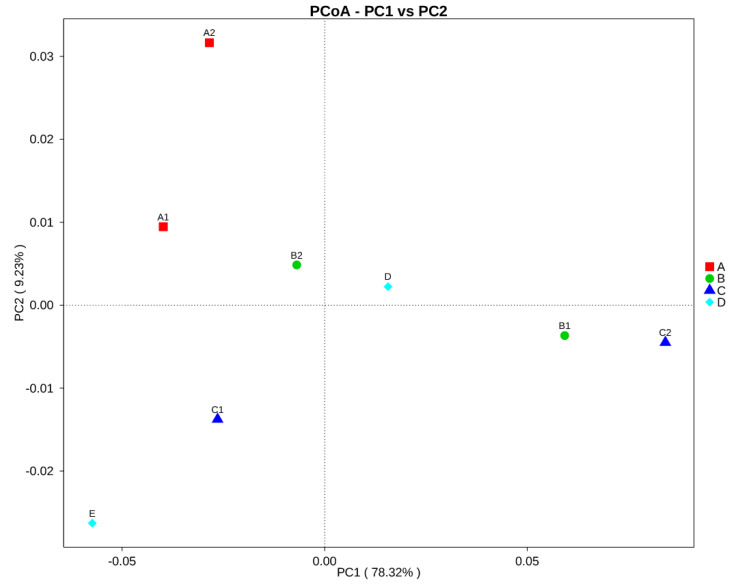
Principal coordinates analysis (PCoA) of the bacterial communities in honey from eight different stingless bee species. Principle coordinate analysis is based on the weighted Unifrac metric for bacterial communities. The red square represents stingless bee from *Heterotrigona* genus, the green dot represents *Trigona* genus, the blue triangle represents *Tetrigona* genus, meanwhile sample D and E represents separate genus which are *Geniotrigona* and *Homotrigona,* respectively.

Based on the relative abundance of OTUs, a phylogeny-based weighted Unifrac principle coordinate analysis, PCoA (Figure 3) showed no clustering points, which indicates the dissimilarity and wide difference in bacterial community among all samples of both same and different stingless bee genera. Therefore, the type of bacterial community present in stingless bee honey is not influenced by the stingless bee taxonomic group up to genus level.

## 4. Discussion

Numerous studies have been conducted on the bacterial community of honeybee species. Several have been conducted on stingless bee species, but only a handful have focused on stingless bee honey. The majority of the studies are narrowly focused on gut bacterial functions related to bee nutrition and their protection against harmful microorganisms [22]. This has lately become a trend in the insect research community. The overlooked branch of study of the stingless bee needs to be further explored, specifically studies that correlate the relationship between bees and humans, such as the antimicrobial benefits of their honey against human pathogens. Based on our results, *H. fimbriata* had the highest antimicrobial activity against the selected bacteria. However, this result is not related to the bacterial diversity of the honey of each stingless bee species, as *H. fimbriata* is not ranked as the highest or lowest in bacterial diversity index component (Table 2). There is no proven result that relates high bacterial diversity with high antimicrobial activity of a substance. However, bacteria from the genus *Lactobacillus* have been recognized as human probiotics since a couple of decades ago for their ability to produce various antimicrobial compounds and other probiotic related substances that lead to its adhesive as well as bactericidal properties [22]. Yet, the scarce or absent antibacterial activity in other SBH dominated by *Lactobacillus* spp. in this study could be due to strain specific production of antimicrobial activity. Several studies have shown that the occurrence of secondary metabolites gene clusters that are responsible for synthesizing antimicrobial compound in *Lactobacillus* spp. depend on the type of strain [23,24,25]. Due to limitations of the taxonomic resolution power of 16S rRNA amplicon sequencing technology [26], this study could not to look at strain differences among SBH. Future work on isolating *Lactobacillus* strains from the targeted SBH may be done to test their antimicrobial activity and further test this hypothesis.

Besides *Lactobacillus*, other factors, such as the acidity of the honey itself, may have contributed partially towards its antimicrobial activity. Of all studied species, stingless bee honey is known to have a low pH, ranging from 3 to 5 [27,28]. This level of acidity may be fatal for many bacteria. Our study showed a lower pH value for each SBH compared to past studies with an average point of 2.3 [28] (Table 1). However, our results indicate little correlation between high acidity with high antimicrobial activity of SBH. The SBH with the lowest antimicrobial activity (*H. erythrogastra*) has indeed the highest acidity (pH 1.82).

One of the factors that may have led to the variant honey bacterial diversity among the studied species is moisture content. High moisture can encourage the growth of bacteria, which is consistent with our results for *T. melanoleuca* that has both high moisture content (43 g/100 g) and high bacterial diversity (H = 2.10, D = 0.47) as proven from our study [28]. At the same time, *G. thoracica* has low moisture content (28.17 g/100 g) and low bacterial diversity (H = 0.85, D = 0.22) [28]. Nonetheless, other factors such as acidity, diastasis, etc. may also contribute to the bacterial diversity composition of honey, and hence should not be omitted. Overall, stingless bee honey is rich with a select set of microorganisms, and thus a more thorough list of contents of the honey we consume is needed. Moreover, knowledge of the honey bacterial community will allow for a broader understanding of the honey maturation process, as certain microorganisms are responsible for its fermentation reaction [29]. Therefore, stingless beekeeping techniques—particularly in honey storage—can be further improved. *Bacillus* can stimulate the fermentation process in honey under aerobic condition in which alcohol and oxygen molecules are converted into acetic acid and water [30,31]. A recent local study has shown that this bacteria was found abundantly in honey produced by *Heterotrigona itama* [32]. However, in our study, *Bacillus* bacteria were only found in significance in sample C2. As shown in Figure 2, *Tetrigona bingami* showed a low relative abundance of only 0.15% (Table S2).

The honey bacterial community of our samples is mainly composed of the Firmicutes phylum, or more specifically, *Lactobacillus* bacteria. *Lactobacillus* bacteria generally have the ability to produce bacteriocins, which acts as an antimicrobial compound to numerous pathogens such as *Escherichia coli*, *Salmonella enterica* and *Candida albicans* [13]. Previous studies has also found the presence of *Lactobacillus* bacteria inside the honey stomach of *Apis mellifera* [33]. The honey stomach is a crop organ inside the bee that serves as a reservoir for the collected nectar and the enzymes inside the crop will act upon the nectar to turn it into honey. Another similar study of a different honey bee species, *Apis dorsata* has also found *Lactobacillus* bacteria inside the honey stomach [34]. However, unlike stingless bee species which have a single dominant bacterial genus, honey bee species always have a pair set of dominant bacteria which is *Lactobacillus* and *Bifidobacterium* [34,35]. The lack of dominant bacterial genera in stingless bee honey may be related to its foraging nature. Stingless bee species do not collect food far from the bee hive, and therefore may only acquire a limited variation of microbe species from the close environment [36]. In addition, stingless bee species have a selective floral preferences and highly acidic honey content (76.3 meq/kg on average) which may contribute to the single dominant bacterial genus occurrence in this study [37]. Only a few selected bacteria have the ability to grow in acidic environment such as *Lactobacillus* sp., hence their omnipresent in SBH [38].

Although *Lactobacillus* bacteria may be acquired from the flowers surrounding stingless bees, there is evidence indicating that some of it originates from the honey bee stomach itself (primary sources) [39]. This may explain the presence of *Lactobacillus* bacteria in the honey of all eight stingless bee species, as the bee also retains honey in the stomach that harbor a few lactic acid bacteria (LAB) for fermentation process [40]. A study has shown a possible positive trend between the prevalence of *Lactobacillus* species in honey microbiome with the productive worker bees. Apparently, worker bees with higher honey production also have more abundant and diverse *Lactobacillus* bacteria [40]. This indicates the natural existence and significance of *Lactobacillus* sp. for honey production generally. However, the study also stresses on the importance of further investigations before correlating both factors as their studied samples size are small and inconsistent. In a slight contrast, a review study by Moran (2015) affirmed that many bees and its product have relatively small bacterial diversity at species-level yet high diversity at strain-level [41]. For example, different colonies or individual bumble bees have different strains of *Gilliamella apicola* bacteria. An individual bee may contain *G. apicola* with the ability to express pectate lyase gene while other individual retain the same species with inability to express one [42].

In our study, seven *Lactobacillus* species were identified as dominant in the studied stingless bee honey. One of the species predominantly and abundantly present in all stingless bee species, proving its native nature. This bacteria is known as *Lactobacillus malefermentans* and was first isolated from beer [43]. Being a member of LAB group, it has the ability to ferment glucose, maltose and inulin. Other important bacterial characteristics such as antibiotic activity are not explored for this bacterial species and this study would be the triggering effect for its further characterization in the future. Another most abundant bacterial species identified from this study is *Lactobacillus wasatchensis*; 30.91% in *Tetrigona apicalis*, 10.59% in *Tetrigona melanoleuca* and 58.74% in *Tetrigona bingami* (Table S2). These bacteria are commonly isolated from cheddar cheese and can cause unwanted cracks on cheese due to late carbon dioxide gas formation [44,45]. The benefit of *L. wasatchensis* to human health is also understudied and need to be further analyzed as indicated by this study.

Meanwhile, *Lactobacillus johnsonii* is a well-studied bacteria with great probiotic advantages for humans. Evidence-based study has shown its probiotic attribute, in particular the inhibition of pathogenic *Helicobacter pylori* adhesion—and its subsequent activity in the human intestine—therefore reducing gastritis symptoms [46]. Aside from that, *L. johnsonii* has potential as marketable probiotic substance due to its great resistance towards gastric acid in stomach [47]. This bacterial species appeared to be abundant in the honey of *Heterotrigona erythrogastra* species (9.52%) (Table S2). The particular knowledge of apprehending which stingless bee honey carries *L. johnsonii* bacteria will enable the traditional medicine practitioner to choose the right honey for treating gastric disease.

The results obtained from this study will carve pathways for future direction in the research of honey derived from stingless bees. For example, scientists can specifically focus on isolating *Lactobacillus* sp. from the honey of *H. fimbriata* which shows high antimicrobial activity. Followed by functional ontology, studies of the isolated bacteria will further reveal its value as probiotic at genome level. This analysis can be conducted via the whole genome sequencing technology. The isolated and specified bacterial species can then be added into health and dietary supplementary products for instance yogurt, fermented milk, probiotics capsule pills, lozenges and the list go on. Such actions have been done on *Lactobacillus acidophilus* isolated from a honey bee species, *Apis cerana indica* in which the bacteria as well as the honey are incorporated into a probiotic honey beverage for non-dairy market purpose [48]. Based on our findings, a few dominant stingless bee honey bacteria such as *Lactobacillus malefermentans*, *Lactobacillus wasatchensis* and *Lactobacillus johnsonii* can be selected for further analysis and perhaps a new probiotic product with intriguing health benefits will emerge.

## 5. Conclusions

In this study, we conducted antimicrobial tests of honey derived from eight different stingless honey bee species to assess their potential as probiotics. The honey from *Homotrigona fimbriata* species showed the highest antimicrobial activity, with inhibition of four of five tested bacteria. However, the antimicrobial activity of SBH will be better assessed against a broader range of microorganisms in future work. The 16S amplicon sequencing technology has also been used to characterize honey bacterial community from the studied stingless bee species and identified a total of eight bacterial phyla, 71 families, 155 genera and 70 species. Honey produced by stingless bee species, *Heterotrigona erythrogastra* and *Tetrigona melanoleuca* are the top two with the highest bacterial diversity compared to other six species. *Lactobacillus malefermentans* may serve as the native dominant bacteria of honey produced by stingless bees due to its consistently dominant presence in all eight stingless bee species. This study has improved the knowledge of stingless bee honey bacterial community at species level. Future works focusing on *Lactobacillus* sp. isolated from *H. fimbriata*’s honey identified from this study can be done in the effort to utilize its probiotic ability in the food technology industry.

## Figures and Tables

**Table 1:** Antimicrobial activity of stingless bee honey against five different bacteria and pH value.

Stingless Bee Honey Samples of Different Species & Concentration		Inhibition Zone Measurement Against Different Bacteria (mm)				
	pH	*S. aureus*	*A. faecalis*	*S. marcescens*	*B. subtilis*	*E. coli*
*H. fimbriata*	2.64 ± 0.17					
50%		28 ± 0.58	21 ± 1.00	18 ± 1.00	9 ± 3.72	12 ± 0.58
25%		19 ± 0.58	15 ± 0.58	12 ± 0.58	0 ± 2.40	11 ± 0.00
12.5%		16 ± 0.58	0 ± 0.00	11 ± 0.00	0 ± 8.59	9 ± 0.58
*T. melanoleuca*	2.89 ± 0.20					
50%		22 ± 0.58	21 ± 1.00	14 ± 0.58	9 ± 0.82	9 ± 1.00
25%		18 ± 0.58	0 ± 0.00	11 ± 1.53	11 ± 9.88	0 ± 0.00
12.5%		0 ± 0.00	0 ± 0.00	10 ± 1.53	0 ± 0.00	0 ± 0.00
*H. itama*	2.57 ± 0.19					
50%		13 ± 0.00	0 ± 0.00	0 ± 0.00	12 ± 0.00	0 ± 0.00
25%		11 ± 0.58	0 ± 0.00	0 ± 0.00	0 ± 0.00	0 ± 0.00
12.5%		10 ± 0.00	0 ± 0.00	0 ± 0.00	0 ± 0.00	0 ± 0.00
*T. apicalis*	2.20 ± 0.21					
50%		13 ± 0.58	0 ± 0.00	0 ± 0.00	12 ± 0.00	0 ± 0.00
25%		9 ± 1.53	0 ± 0.00	0 ± 0.00	0 ± 0.00	0 ± 0.00
12.5%		8 ± 0.58	0 ± 0.00	0 ± 0.00	0 ± 0.00	0 ± 0.00
*L. terminata*	2.36 ± 0.20					
50%		11 ± 0.58	0 ± 0.00	0 ± 0.00	12 ± 1.53	0 ± 0.00
25%		10 ± 0.58	0 ± 0.00	0 ± 0.00	9 ± 0.58	0 ± 0.00
12.5%		0 ± 0.00	0 ± 0.00	0 ± 0.00	0 ± 0.00	0 ± 0.00
*G. thoracica*	1.95 ± 0.13					
50%		11 ± 1.00	0 ± 0.00	0 ± 0.00	0 ± 0.00	0 ± 0.00
25%		9 ± 1.53	0 ± 0.00	0 ± 0.00	0 ± 0.00	0 ± 0.00
12.5%		0 ± 0.00	0 ± 0.00	0 ± 0.00	0 ± 0.00	0 ± 0.00
*T. binghami*	2.19 ± 0.19					
50%		10 ± 0.00	0 ± 0.00	0 ± 0.00	0 ± 0.00	0 ± 0.00
25%		0 ± 0.00	0 ± 0.00	0 ± 0.00	0 ± 0.00	0 ± 0.00
12.5%		0 ± 0.00	0 ± 0.00	0 ± 0.00	0 ± 0.00	0 ± 0.00
*H. erythrogastra*	1.82 ± 0.l2					
50%		0 ± 0.00	0 ± 0.00	0 ± 0.00	9 ± 1.00	0 ± 0.00
25%		0 ± 0.00	0 ± 0.00	0 ± 0.00	0 ± 0.00	0 ± 0.00
12.5%		0 ± 0.00	0 ± 0.00	0 ± 0.00	0 ± 0.00	0 ± 0.00

**Table 2:** Richness and diversity estimation of the bacterial community in honey from eight different stingless bee species (A1—*Heterotrigona itama*; A2—*Heterotrigona erythrogastra*; B1—*Tetrigona apicalis*; B2—*Lepidotrigona terminata*; C1—*Tetrigona melanoleuca*; C2—*Tetrigona bingami*; D—*Geniotrigona thoracica*; E—*Homotrigona fimbriata*).

Sample Name	Number of Bacterial OTU	Shannon Index (H)	Simpson Index (D)
A2	158	2.18	0.53
C1	205	2.10	0.47
A1	131	2.00	0.47
E	149	1.96	0.42
C2	51	1.60	0.56
B2	106	1.18	0.25
B1	20	1.76	0.68
D	28	0.85	0.22

## Supplementary Materials

The following are available online at https://www.mdpi.com/2075-4450/11/8/500/s1, Table S1: Percentage Abundance of Top Seven OTU Bacteria (OTU%). Table S2: Percentage Abundance of Top Bacterial Genus (OTU%). Figure S1: Rank abundance curve of bacterial composition in honey from eight different stingless bee species.
