# Supplementary material for: Stingless Bee Honey: Evaluating Its Antibacterial Activity and Bacterial Diversity
Source: Insects. 2020 Aug 4;11(8):500. doi: 10.3390/insects11080500 (PMC7469184; doi:10.3390/insects11080500)
Supplement: Supplementary file 1 [file insects-11-00500-s001.zip › insects-821259-supplementary/supp/Figure S1 (1).pdf]

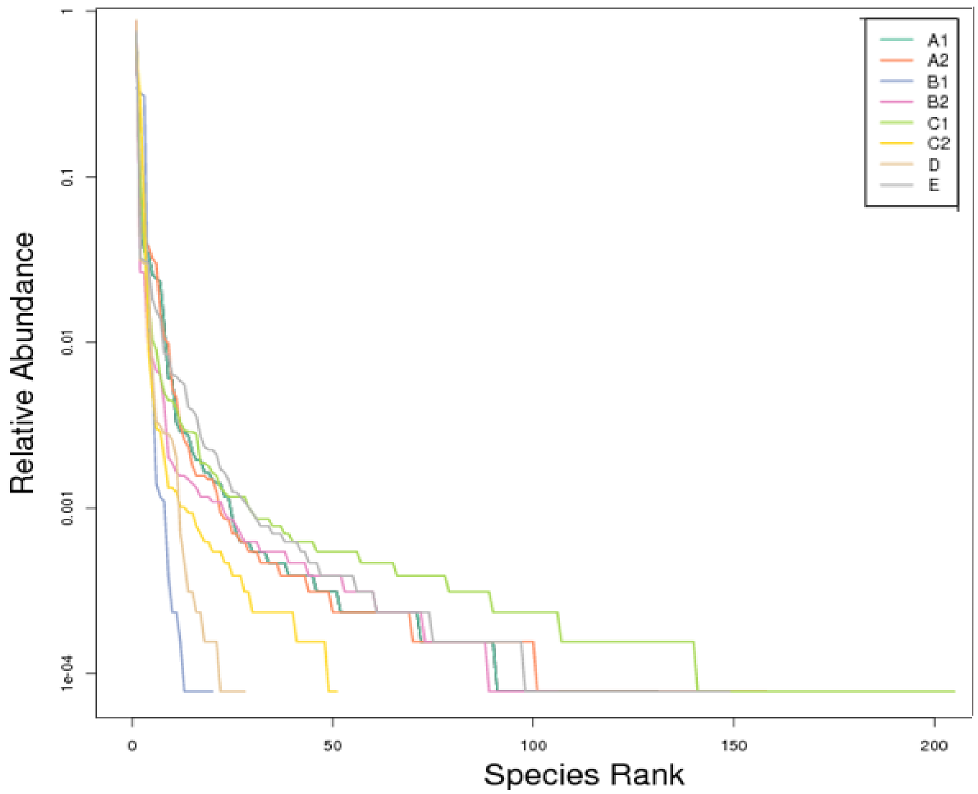

Figure S1. Rank abundance curve of bacterial composition in honey from eight different stingless bee species. Steep line graphs represents low bacterial diversity due to its low number of species rank and low relative abundance.
